# Pediatric campylobacteriosis in northern Taiwan from 2003 to 2005

**DOI:** 10.1186/1471-2334-8-151

**Published:** 2008-10-31

**Authors:** Ji-Rong Yang, Ho-Sheng Wu, Chuen-Sheue Chiang, Jung-Jung Mu

**Affiliations:** 1 Research and Diagnostic Center, Centers for Disease Control, Department of Health, Taipei, Taiwan; 2 School of Medical Laboratory Science and Biotechnology, Taipei Medical University, Taipei, Taiwan; 3 Center of General Education, National Taipei College of Nursing, Taipei, Taiwan

## Abstract

**Background:**

There has been a marked increase in the incidence of, and concern regarding, human *Campylobacter jejuni *and *C. coli *infections worldwide during the last decade. As the highest infectious disease control apparatus in Taiwan, we aimed to describe the character of *Campylobacter *isolates from infected children, as well as basic information about the patients, from December 2003 to February 2005.

**Methods:**

A total of 894 fecal specimens were collected by several clinics and hospitals from children who had diarrhea, followed by plating onto selective media. Drug susceptibility test of the isolates from these specimens were conducted by disc diffusion method and their serotypes were also studied using commercial antisera made in Japan.

**Results:**

The isolation rate of *Campylobacter *during these 15 months was 6.8% and was higher in winter (11.1%) than in other seasons. *C. jejuni *was the most prevalent (95.1%) species in northern Taiwan, comparable to other developed countries. Among the 61 *Campylobacter *isolates, most were resistant to tetracycline (93.4%), nalidixic acid (91.8%), ciprofloxacin (90.2%), and ampicillin (85.5%). Erythromycin-resistant isolates represented 3.3% of all isolates, suggesting that this drug may be the first choice for treatment. The serotypes of the 61 isolates were demonstrated and only 41.4% were typable.

**Conclusion:**

In this study, the Taiwan CDC provided an epidemiological analysis of *Campylobacter *infection, including the isolation rate, age, seasonal distribution, antimicrobial drug susceptibility patterns, and serotypes of the isolates from pediatric patients in northern Taiwan from 2003 to 2005.

## Background

Infection with thermophilic *Campylobacter *species, especially *C. jejuni *and *C. coli*, is one of the most common causes of bacterial diarrhea in humans worldwide [1-3]. The pathogen is also part of the normal intestinal flora of domestic and wild animals [4,5]. Infections caused by this pathogen are usually sporadic, occurring in the summer months and early fall in many countries and usually following the ingestion of improperly handled foods. Among developing countries in Asia, the incidence of diseases caused by *Campylobacter *is much higher than that in developed countries [4]. In Thailand, the overall isolation rate of *Campylobacter *from diarrheal children under year 5 was 13.0% [6]. This rate was also present about 12.1% in Laos that *C. jejuni *together with *C. coli *occupied 7.1% and 4% of enteric infection in children aged < 1 year and 1–5 years, respectively [7]. In the United States, the incidence of campylobacteriosis was 12.71 per 100,000 population and has often exceeded those of salmonellosis and shigellosis during the past years [8,9]. *Campylobacter *spp. are also the major pathogens, along with enterotoxigenic *Escherichia coli*, responsible for traveler's diarrhea [4]. The symptoms associated with campylobacteriosis range from mild enteritis with or without bloody diarrhea, fever, abdominal pain to severe invasive diseases, and are sometimes more severe than *Salmonella *and *Shigella *infections [10]. In addition, *Campylobacter *infection is associated with the development of Guillain-Barre syndrome, which is an autoimmune disorder of the peripheral nervous system and characterized by acute flaccid paralysis [11].

Most *Campylobacter *infections are self-limited, and treatment is usually supportive. Antimicrobial therapy should still be considered if prolonged or severe symptoms occurred. Macrolides (erythromycin) and fluoroquinolones (ciprofloxacin) are recommended as first and alternative choices of therapy, respectively [12,13]. However, a rapid increasing proportion of *Campylobacter *strains have developed resistance to the fluroquinolones and macrolides in the past decade, especially in Southeast Asia, the United States and Europe. This illustrated the antimicrobial resistance of *Campylobacter *has emerged worldwide [14].

National surveillance programs that facilitate monitoring of sporadic and outbreak cases of human campylobacteriosis in many developed countries, such as the United States and members of the European union, are available [4]. In contrast, the information of the incidence of campylobacteriosis in developing countries is limited, and most are from laboratory-based data. Therefore, the rate of *Campylobacter *infection in those countries may be underestimated [4,15,16]. In 1998, Lin et al. has reported campylobacteriosis in diarrheal children in central Taiwan with 2.5% isolation rate and showed more prevalent in winter [16]. As the highest health and infectious diseases control apparatus in Taiwan, we described here the character of *Campylobacter *isolates from infected children, including the isolation rate, seasonal distribution, antimicrobial drug susceptibility patterns, and serotypes as well as basic information about the patients, including age, gender, and symptoms, in northern Taiwan from December 2003 to February 2005.

## Methods

### Collection and transportation of clinical specimens

The study population in the present study consists of children under age 17 in northern Taiwan with loose, watery or bloody diarrhea and divides into 5 age-groups as follows: (1) < 4 years old (infant stage), (2) 4–7 (kindergarten stage), (3) 7–10 (elementary school stage 1), (4) 10–13 (elementary school stage 2) and (5) > 13 (junior high school stage and above). The fecal specimens were collected from more than 30 clinics and hospitals in northern Taiwan, including Taipei, Taoyuan, Hsin-Chu, Yilan, and Keelung. All the samples which were transported in Cary-Blair transport medium (BD Diagnostic Systems, Sparks, MD) or buffered glycerol saline (Creative Microbiologicals, Ltd., Taipei, Taiwan) were previously tested the presence of other enteric pathogens such as *Shigella *spp., *Salmonella *spp. and *Vibrio *spp. Specimens free of the above pathogens were chosen in our study. Therefore, mixed infection with multiple pathogens in this study has been excluded. A total of 894 clinical specimens from different patients gathered between December 2003 and February 2005 were chosen.

### Enrichment or plating medium selection

After arrival at our laboratory, the specimens were directly inoculated onto two prepared selective media (usually delay 1–7 days between stool collection and plating): modified cefoperazone charcoal deoxycholate agar (mCCDA, Creative Microbiologicals, Ltd., Taipei, Taiwan) and charcoal-containing selective medium (CSM, BD Diagnostic Systems, Sparks, MD), followed by incubation at 42°C for 48 hours in a microaerobic atmosphere containing 5% O_2_, 10% CO_2_, and 85% N_2 _generated by CampyPak (BD Diagnostic Systems, Sparks, MD). If enrichment was needed to recover low numbers of organisms due to delayed transport to the laboratory, swabs or stools were first inoculated into brain heart infusion broth (BHI, Creative Microbiologicals, Ltd., Taipei, Taiwan) and *Campylobacter *enrichment broth, each of which contained cefoperazone (8 mg/L), amphotericin B (5 mg/L), teicoplanin, and 5% lysed horse blood, followed by incubation at 37°C for 48 hours. Finally, the broth suspensions were inoculated onto mCCDA or CSM agar for another 48 hours.

### Phenotypic characterization of isolates

For further confirmation of *Campylobacter*, suspected colonies, which were morphologically smooth, gray, flat, and moist, were sub-cultured from each plate onto fresh blood agar plates. They were checked by Gram staining and several biochemical reactions including oxidase production, catalase production, and sodium hippurate hydrolysis. Oxidase- and catalase-positive colonies exhibiting a characteristic Gram stain appearance (e.g., Gram-negative, curved rods) could be reported as *Campylobacter *spp. Hydrolysis of sodium hippurate is the major identification test used for *C. jejuni*. *Campylobacter *species giving a positive hippurate hydrolysis result could be reported as C. *jejuni*, with no other additional tests required. Isolates giving a negative result for hippurate hydrolysis were further identified by PCR in order to distinguish some isolates of *C. jejuni *that express the hippuricase gene (*hipO*), but are negative for the hippurate hydrolysis phenotype from *C. coli*, another important species of *Campylobacter *that lacks hippuricase activity, as described below.

### Conventional PCR identification

To confirm isolates that were negative for hippurate hydrolysis activity as *C. jejuni *or *C. coli*, conventional PCR was conducted. DNA was extracted from bacterial suspensions by boiling at 100°C for 15 min. Two primer pairs [17,18] CJF (5'-ACTTCTTTATTGCTTGCTGC-3'), CJR (5'-GCCACAACAAGTAAAGAAGC-3') and COL1 (5'-ATGAAAAAATATTTAGTTTTTGCA-3'), COL2 (5'-ATTTTATTATTTGTAGCAGCG-3') each were used to amplify the *hipO *gene from *C. jejuni *and the *ceuE *gene from *C. coli*, respectively. PCR was performed using conditions described, as previously reported [17,18].

### Antimicrobial susceptibility test

To determine the susceptibility of *Campylobacter *isolates to antimicrobial agents, they were suspended in Mueller-Hinton broth (Creative Microbiologicals, Ltd., Taipei, Taiwan) to the concentration of McFarland No. 0.5 and bacterial cells were inoculated on Mueller-Hinton agar plates (Creative Microbiologicals, Ltd., Taipei, Taiwan) containing 5% sheep blood after incubation for 48 hours in a microaerobic atmosphere. Discs containing ampicillin (10 μg), chloramphenicol (30 μg), nalidixic acid (30 μg), ciprofloxacin (5 μg), cephalothin (30 mg), clindamycin (2 μg), erythromycin (15 μg), gentamicin (10 μg), streptomycin (10 μg), and tetracycline (30 μg) (Oxoid, Hampshire, UK) were placed on the inoculated plates. The plates were then incubated at 42°C for 48 hours in a microaerobic atmosphere. Interpretation of the susceptibility test, including susceptible (S), intermediate (I) and resistant (R), was carried out (Available from ) [19,20]. *Staphylococcus aureus *ATCC 29213, *Escherichia coli *ATCC 25922, and *Pseudomonas aeruginosa *ATCC 27853 were used as positive controls.

### Serotyping of somatic antigen

For serotyping of *C. jejuni*, a commercial antisera kit (Denka Seiken, Tokyo, Japan) designed to detect the heat-stable antigen was utilized by the passive hemagglutination method (PHA). For preparation of the sensitized bacterial antigen solution, the bacteria and 0.25 ml of each extraction solution provided in the kit were mixed. To prepare sensitized red blood cells for use in the PHA test, 0.5 ml of each bacterial antigen was incubated with fixed chick red blood cells at 37°C for 30 min. The PHA test was conducted by placing one drop of each antiserum and 25 μl of sensitized red blood cells into microplate wells and incubating for 30 min. Examination for agglutination in each well was utilized to determine the serotypes of the C. *jejuni *isolates.

### Ethical review

The sentinel surveillance for diarrhea syndrome is one of the national disease surveillance systems established by Taiwan CDC. Sentinel physicians in individual hospitals, clinics and medical centers reported outpatients suspected of catching diarrhea and sent specimens for enteric bacterial pathogens identification. The present study was carried out through sentinel surveillance and reviewed by Taiwan CDC, which does not require oversight by an ethics committee.

## Results

### *Campylobacter *isolates from clinical fecal specimens

**Figure 1:**
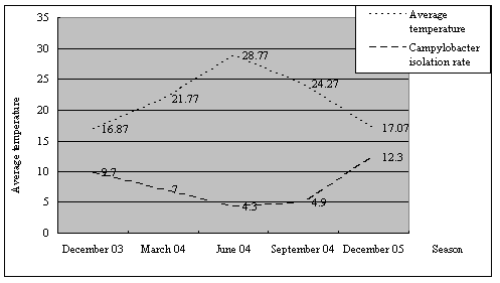
Relationship between the *Campylobacter *isolation rate and the average temperature of each season.

To study the prevalence rate of *Campylobacter *in children in northern Taiwan, a total of 894 fecal specimens from different diarrheal patients were tested for *Campylobacter*. Sixty-one isolates (61/894, 6.8%) including 58 *C. jejuni *(58/61, 95.1%) and 3 *C. coli *(3/61, 4.9%) isolates were identified by direct plating, followed by biochemical reactions and PCR. Two selective culture media (mCCDA and CSM) were used and the recovery rates from both were the same (data not shown). During the 15 months of the study, the highest and lowest positive rates were observed in February 2005 (4/22, 18.2%) and August 2004 (1/82, 1.2%), respectively. Most isolates were recovered in winter (December to February, 25/225, 11.1%), followed by spring (March to May, 15/214, 7.0%), autumn (September to November, 11/225, 4.9%), and summer (June to August, 10/230, 4.3%). The positive rate of *Campylobacter *and its relationship with the average temperature during each month are shown in Table 1 and Figure 1, respectively.

**Table 1:** Positive rate of campylobacteriosis for each month from December 2003 to February 2005

Year	03	04												05	
			
Month	12	1	2	3	4	5	6	7	8	9	10	11	12	1	2
No. of specimens	26	45	32	91	49	74	69	79	82	99	70	56	60	40	22
No. of positives	4	5	1	4	6	5	4	5	1	2	5	4	7	4	4
Positive rate (%)	15.4	11.1	3.1	4.4	12.2	6.8	5.8	6.3	1.2	2.0	7.1	7.1	11.7	10.0	18.2

### Basic information about infected patients

**Figure 2:**
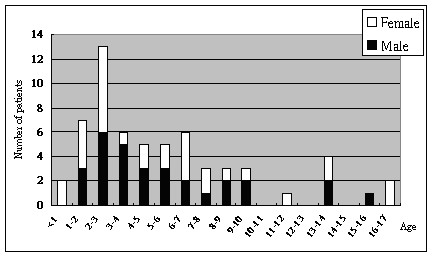
Distribution of *Campylobacter *isolates according to age and sex of the 61 patients.

Of the 61 *Campylobacter *isolates, 29 (47.5%) and 32 (52.5%) each were gathered from male and female children, respectively (Figure 2). The age of these 61 patients ranged from 10 months to 17 years old (Figure 2). We further divided them into 5 age-groups according to the school age (see Methods). Children in the < 4 group had the highest positive rate (28/61, 45.9%), followed by the 4–7 group (16/61, 26.2%), group 7–10 (9/61, 14.8%), group > 13 (7/61, 11.5%) and group 10–13 (1/61, 1.6%). Bloody diarrhea is a classical symptom in children with campylobacteriosis [21]. Of the infected children, 25 out of 61 (41%) had bloody diarrhea (Table 2). Most of these 25 children belonged to the < 4 age group (14/25, 56%), followed by group 4–7 (4/25, 16%), group 7–10 (3/25, 12%), group > 13 (3/25, 12%) and group 10–13 (1/25, 4%). Thirty-nine out of 61 (39/61, 63.9%) presented with fever, and most of these 39 children belonged to the < 4 age group (14/39, 35.9%), followed by group 4–7 (9/39, 23.1%), group 7–10 (9/39, 23.1%), group > 13 (6/39, 15.4%), and group 10–13 (1/39, 2.5%).

**Table 2:** Symptoms of the 61 patients in the five age groups

Age group (year)	No. of patients	No. (%) of Bloody diarrhea	No. (%) of fever	No. (%) of times of diarrhea/day	
				
				1~4	> 4
< 4	28	14 (50)	14 (50)	22 (78.6)	7 (25)
4–7	16	4 (25)	9 (56.3)	8 (50)	2 (12.5)
7–10	9	3 (33.3)	9 (100)	8 (88.9)	5 (55.6)
10–13	1	1 (100)	1 (100)	1 (100)	0
> 13	7	3 (42.9)	6 (85.8)	6 (85.7)	2 (28.6)

Total	61	25 (41)	39 (63.9)	45 (73.8)	16 (26.2)

### Antimicrobial susceptibility

The Clinical and Laboratory Standards Institute (CLSI) has approved the agar dilution method as a standard susceptibility testing method for *Campylobater*. Due to the time-consuming procedures, it is rarely carried out in routine diagnostic laboratories. The easier and less expensive disc diffusion method represents a good correlation while comparing with agar-based dilution method and provides an alternative choice for antibiotic susceptibility test of *Campylobacter *[20,22]. Since the disc diffusion method was found to be a reliable and easy tool for monitoring the prevalence of *Campylobacter *strains, we applied this method for antimicrobial susceptibility test in this study.

The results of the susceptibility testing for 61 *Campylobacter *isolates are shown in Table 3. All *C. jejuni *and *C. coli *isolates in this study were resistant to cephalothin (100%), and most showed resistance to the four other drugs tested, including tetracycline (93.4%), nalidixic acid (91.8%), ciprofloxacin (90.2%), and ampicillin (85.5%). Five out of the ten antimicrobial agents tested, including streptomycin, chloramphenicol, gentamicin, clindamycin, and erythromycin, tended to result in a lower resistance rate as listed in Table 3. Cross-resistance between nalidixic acid and ciprofloxacin was found in all quinolone-resistant isolates.

**Table 3:** Antimicrobial drug resistance of the 61 *Campylobacter *isolates

Antibiotic drug (μg)	Number of resistant (%)
	
	*Campylobacter *n = 61
Ampicillin (10)	52 (85.2)
Cephalothin (30)	61 (100)
Chloramphenicol (30)	10 (16.4)
Ciprofloxacin (5)	55 (90.2)
Clindamycin (2)	3 (4.9)
Erythromycin (15)	2 (3.3)
Gentamicin (10)	6 (9.8)
Nalidixic Acid (30)	56 (91.8)
Tetracycline (30)	57 (93.4)
Streptomycin (10)	15 (24.6)

### Serotyping of the 58 *C. jejuni *isolates

For serotyping, 24 out of the 58 *C. jejuni *isolates (24/58, 41.4%) were typable and belonged to 9 different serotypes according to Penner's scheme (Table 4). Five of the above 9 serotypes contained only one isolate and the other four types contained two to eight isolates. The most prevalent serotypes among these isolates were Penner's type 2 (group B), followed by type 6,7 (group F) and type 11 (group J). Thirty-four (58.6%, 34/58) strains were defined as "untypable", including 14 (41.2%, 14/34) strains that failed to agglutinate in the presence of any of the antisera used in the present study and 20 that reacted with two or more types of antisera. Serotyping using Penner's scheme was sufficient for identification of about half of the human clinical strains.

**Table 4:** Penner's serotypes of the 58 *C. jejuni *isolates

Penner's serotype	No. of isolates (%)
Penner's type 2	8 (13.9)
Penner's type 3	2 (3.5)
Penner's type 4	1 (1.7)
Penner's type 5	1 (1.7)
Penner's type 6	5 (8.6)
Penner's type 7	1 (1.7)
Penner's type 10	1 (1.7)
Penner's type 11	4 (6.9)
Penner's type 14	1 (1.7)
Untypable	34 (58.6)
Total	58 (100)

## Discussion

Over the past years, the limited number of reports regarding *Campylobacter *infection may lead to the possible under-estimation of the incidence of campylobacteriosis in Taiwan. Due to heightened public concern, we have already set up a *Campylobacter *reference laboratory at the Taiwan CDC to be responsible for the surveillance study since 2003. In this study, we provided an epidemiological analysis of *Campylobacter *infection, including the isolation rate, age, seasonal distribution, antimicrobial drug susceptibility patterns, and serotypes of the isolates from pediatric patients in northern Taiwan from 2003 to 2005.

Among the 894 clinical specimens obtained from children tested for campylobacteriosis, 61 isolates (6.8%) were recovered in our study. This percentage was comparable to that obtained by van Hees et al. at 6.3% [23]. In addition, *Campylobacter *may present much more frequently in northern Taiwan (6.3%) while compare to the report of campylobacteriosis in central Taiwan by Lin et al. (2.5%) [16]. The age distribution within the 61 patients peaked in the < 4 group (41%, Figure 2) and this was also observed by Feizabadi et al. in Iran and Lin et al. in central Taiwan [16,24]. Therefore, the preschool children may have a relatively high risk of being infected by this important pathogen.

There are many species among *Campylobacter *cause human diarrhea, such as *C. jejuni*, *C. coli*, *C. fetus*, *C. lari*, and *C. upsaliensis*. *Campylobacter jejuni *and *C. coli *are the only species (95.1% and 4.9%, respectively) isolated from specimens examined in our study. This suggests that *Campylobacter *infection of children in northern Taiwan is similar to other countries [25,26]. Feizabadi et al. and Gallay et al. have both reported the rates of *C. jejuni *and *C. coli *in human specimens in Iran and France [5,24], respectively. The percentage of *C. jejuni *in their isolated strains was 85.8% and 76.2% and that of *C. coli *was 14.2% and 17.2%, respectively. In these two studies, *C. coli *seemed to be present at a higher percentage compared to our study. To obtain the true rates of *C. jejuni *and *C. coli*, both groups concluded that PCR method is needed on the classification of the hippurate hydrolysis negative strains at the genetic level [17,18]. In our study, all three *hippuricase *negative isolates were confirmed as *C. coli *by PCR. Our data reflect the reliable rates of *C. jejuni *and *C. coli *in northern Taiwan.

According to the results of the seasonal analysis in our study (Figure 1), *Campylobacter *occupied a relative higher percentage in the winter of 2003 (10/103, 9.7%) and 2004 (15/122, 12.3%) (winter *vs*. summer, 95% CI = 0.0190~0.1162), differing from that observed among warmer months such as summer in other reports (10/230, 4.3%) [27]. This seasonal trend was also observed by Lin et al. in central Taiwan and by van Hees et al., who reported the percentage of *Campylobacter *enteritis as 35.2% in winter, 24.1% in summer, and 8.2% in winter, 4.3% in summer, respectively [16,23],. The mechanism which influenced on the different seasonal trend of campylobacteriosis in Taiwan needs to be further studied.

Concern regarding the antimicrobial resistance of human *Campylobacter *isolates has increased in the clinical treatment of patients with campylobacteriosis. Our results revealed that a higher proportion of isolates are resistant to tetracycline, nalidixic acid, ciprofloxacin (Table 3) in comparison with that in NARMS 2004 by the US CDC (93.4%, 91.8% and 90.2% vs 46.1%, 19.6%, and 19.0%, respectively) [28]. Another study was performed by Li et al. in southern Taiwan [29]. The three highest rates of resistance were also observed to be against tetracycline (92.4%), nalidixic acid (80.6%), and ciprofloxacin (57%), and the three lowest rates of resistance were against gentamicin (10.8%), erythromycin, (18.3%) and clindamycin (18.3%). Gallay et al. also reported the resistance rates of human *Campylobacter *isolates in France to tetracycline, ciprofloxacin, and ampicillin as 32.9%, 28.1%, and 34.2%, respectively. The rates of the above three drugs seem to be significantly lower than that of those in northern Taiwan. Erythromycin has been the recommendation of choice for treatment of campylobacteriosis in clinical therapy [30]. To date, the resistance rate reported for erythromycin has varied widely [5]. In the present study, there were only 2 (3.3%) isolates that demonstrated a resistant pattern. According to our results for the susceptibility test, erythromycin and clindamycin may be the best choices to treat patients with campylobacteriosis in northern Taiwan.

As we know, *Campylobacter *could cause not only enteritis but also other disease like GBS. *Campylobacter *isolates in different serotypes may be the agents of the different symptoms (eg. Penner's HS:19 for GBS). Serotyping, therefore, has the benefit for the epidemiologic investigation of *Campylobacter*. The distribution of *C. jejuni *serotypes in northern Taiwan was evaluated in the present study (Table 4) since no other reports were available for the past years. Our results revealed that *Campylobacter *isolates in northern Taiwan were clustered into 9 Penner's serotypes and 58.6% remained untypable. This observation may be explained the presence of different *Camylobacter *serotypes in Taiwan from those in Japan. In the future, homemade antisera that recognize the serotypes of *Campylobacter *present in Taiwan may be required to gain more complete information about the character of these isolates.

## Conclusion

The Taiwan CDC provided a complete epidemiological analysis of *Campylobacter *infection, including the isolation rate, age, seasonal distribution, antimicrobial drug susceptibility patterns, and serotypes of the isolates from pediatric patients in northern Taiwan from 2003 to 2005.

## Competing interests

The authors declare that they have no competing interests.

## Authors' contributions

JRY carried out the experiments, complied the results and wrote the manuscript. HSW participated in the design and CSC and JJM helped to revise the manuscript. All authors approved the final version of the manuscript.

## Pre-publication history

The pre-publication history for this paper can be accessed here:



## References

[B1] Allos BM (2001). *Campylobacter jejuni *Infections: update on emerging issues and trends. Clin Infect Dis.

[B2] Hall G, Kirk MD, Becker N, Gregory JE, Unicomb L, Millard G, Stafford R, Lalor K (2005). Estimating foodborne gastroenteritis, Australia. Emerg Iinfect dis.

[B3] Nachamkin I, Blaser MJ (2000). *Campylobacter*.

[B4] Coker AO, Isokpehi RD, Thomas BN, Amisu KO, Obi CL (2002). Human campylobacteriosis in developing countries. Emerg Iinfect dis.

[B5] Gallay A, Prouzet-Mauleon V, Kempf I, Lehours P, Labadi L, Camou C, Denis M, de Valk H, Desenclos JC, Megraud F (2007). *Campylobacter *antimicrobial drug resistance among humans, broiler chickens, and pigs, France. Emerg Iinfect dis.

[B6] Taylor DN, Perlman DM, Echeverria PD, Lexomboon U, Blaser MJ (1993). *Campylobacter *immunity and quantitative excretion rates in Thai children. J infect dis.

[B7] Yamashiro T, Nakasone N, Higa N, Iwanaga M, Insisiengmay S, Phounane T, Munnalath K, Sithivong N, Sisavath L, Phanthauamath B (1998). Etiological study of diarrheal patients in Vientiane, Lao People's Democratic Republic. J Clin Microbiol.

[B8] Altekruse SF, Stern NJ, Fields PI, Swerdlow DL (1999). *Campylobacter jejuni *– an emerging foodborne pathogen. Emerg Iinfect dis.

[B9] CDC (2007). Preliminary FoodNet data on the incidence of infection with pathogens transmitted commonly through food – 10 states, 2006. MMWR.

[B10] Coker AO, Dosunmu-Ogunbi O (1985). Gastroenteritis due to *Campylobacter jejuni *in Lagos, Nigeria. Cent Afr J Med.

[B11] Takahashi M, Koga M, Yokoyama K, Yuki N (2005). Epidemiology of *Campylobacter jejuni *isolated from patients with Guillain-Barre and Fisher syndromes in Japan. J Clin Microbiol.

[B12] Engberg J, Aarestrup FM, Taylor DE, Gerner-Smidt P, Nachamkin I (2001). Quinolone and macrolide resistance in *Campylobacter jejuni *and *C. coli*: resistance mechanisms and trends in human isolates. Emerg Iinfect dis.

[B13] Hakanen AJ, Lehtopolku M, Siitonen A, Huovinen P, Kotilainen P (2003). Multidrug resistance in *Campylobacter jejuni *strains collected from Finnish patients during 1995–2000. J Antimicrob Chemother.

[B14] Isenbarger DW, Hoge CW, Srijan A, Pitarangsi C, Vithayasai N, Bodhidatta L, Hickey KW, Cam PD (2002). Comparative antibiotic resistance of diarrheal pathogens from Vietnam and Thailand, 1996–1999. Emerg Iinfect dis.

[B15] Chyou SC, Leu YJ, Huang FY, Lee HC, Yang DI (1988). An etiological study of infectious diarrhea in infants and children in Taipei area. Zhonghua Minguo xiao er ke yi xue hui za zhi [Journal].

[B16] Lin CW, Yin PL, Cheng KS (1998). Incidence and clinical manifestations of *Campylobacter *enteritis in central Taiwan. Zhonghua yi xue za zhi = Chinese medical journal; Free China ed.

[B17] Wang G, Clark CG, Taylor TM, Pucknell C, Barton C, Price L, Woodward DL, Rodgers FG (2002). Colony multiplex PCR assay for identification and differentiation of *Campylobacter jejuni*, *C. coli*, *C. lari*, *C. upsaliensis*, and *C. fetus *subsp. *fetus*. J Clin Microbiol.

[B18] Gonzalez I, Grant KA, Richardson PT, Park SF, Collins MD (1997). Specific identification of the enteropathogens *Campylobacter jejuni *and *Campylobacter coli *by using a PCR test based on the *ceuE *gene encoding a putative virulence determinant. J Clin Microbiol.

[B19] Pigrau C, Bartolome R, Almirante B, Planes AM, Gavalda J, Pahissa A (1997). Bacteremia due to *Campylobacter *species: clinical findings and antimicrobial susceptibility patterns. Clin Infect Dis.

[B20] Miflin JK, Templeton JM, Blackall PJ (2007). Antibiotic resistance in *Campylobacter jejuni *and *Campylobacter coli *isolated from poultry in the South-East Queensland region. J Antimicrob Chemother.

[B21] Blaser MJ, Wells JG, Feldman RA, Pollard RA, Allen JR (1983). *Campylobacter *enteritis in the United States. A multicenter study. Ann Intern Med.

[B22] Senok A, Yousif A, Mazi W, Sharaf E, Bindayna K, Elnima el A, Botta G (2007). Pattern of antibiotic susceptibility in *Campylobacter jejuni *isolates of human and poultry origin. Jpn J Infect Dis.

[B23] van Hees BC, Veldman-Ariesen MJ, de Jongh BM, Tersmette M, van Pelt W (2007). Regional and seasonal differences in incidence and antibiotic resistance of *Campylobacter *from a nationwide surveillance study in The Netherlands: an overview of 2000–2004. Clin Microbiol Infect.

[B24] Feizabadi MM, Dolatabadi S, Zali MR (2007). Isolation and drug-resistant patterns of *Campylobacter *strains cultured from diarrheic children in Tehran. Jpn J Infect Dis.

[B25] Endtz HP, Ruijs GJ, Zwinderman AH, Reijden T van der, Biever M, Mouton RP (1991). Comparison of six media, including a semisolid agar, for the isolation of various *Campylobacter *species from stool specimens. J Clin Microbiol.

[B26] Gaudreau C, Gilbert H (1997). Comparison of disc diffusion and agar dilution methods for antibiotic susceptibility testing of *Campylobacter jejuni *subsp. *jejuni *and *Campylobacter coli*. J Antimicrob Chemother.

[B27] Nichols GL (2005). Fly transmission of *Campylobacter *. Emerg Iinfect dis.

[B28] CDC (2004). National antimicrobial resistance monitoring system: enteric bacteria annual report.

[B29] Li CC, Chiu CH, Wu JL, Huang YC, Lin TY (1998). Antimicrobial susceptibilities of *Campylobacter jejuni *and *coli *by using E-test in Taiwan. Scand J Infect Dis.

[B30] Williams MD, Schorling JB, Barrett LJ, Dudley SM, Orgel I, Koch WC, Shields DS, Thorson SM, Lohr JA, Guerrant RL (1989). Early treatment of *Campylobacter jejuni *enteritis. Antimicrob Agents Chemother.

